# Radio-Enhancing Properties of Bimetallic Au:Pt Nanoparticles: Experimental and Theoretical Evidence

**DOI:** 10.3390/ijms20225648

**Published:** 2019-11-12

**Authors:** Daniela Salado-Leza, Ali Traore, Erika Porcel, Diana Dragoe, Antonio Muñoz, Hynd Remita, Gustavo García, Sandrine Lacombe

**Affiliations:** 1 Institut des Sciences Moléculaires d’Orsay (UMR 8214) CNRS, Université Paris-Saclay, Université Paris Sud, 91405 Orsay, France; daniela.salado@conacyt.mx (D.S.-L.); erika.porcel@u-psud.fr (E.P.); 2 Cátedras CONACyT, Universidad Autónoma de San Luis Potosí, Facultad de Ciencias Químicas, Av. Dr. Manuel Nava 6, Zona Universitaria, San Luis Potosí 78210, S.L.P., Mexico; 3 Instituto de Física Fundamental, Consejo Superior de Investigaciones Científicas (CSIC), Serrano 113-bis, 28006 Madrid, Spain; ali_traore@hotmail.fr (A.T.); g.garcia@csic.es (G.G.); 4 Institut de Chimie Moléculaire et des Matériaux d’Orsay (UMR 8182) CNRS, Université Paris Saclay, Université Paris Sud, 91405 Orsay, France; diana.dragoe@u-psud.fr; 5 Centro de Investigaciones Energéticas, Medioambientales y Tecnológicas (CIEMAT), Avda. Complutense 22, 28040 Madrid, Spain; antonio.roldan@ciemat.es; 6 Laboratoire de Chimie Physique (UMR 8000) CNRS, Université Paris Saclay, Université Paris Sud, 91405 Orsay, France; hynd.remita@u-psud.fr

**Keywords:** core-shell gold–platinum nanoparticles, radio-enhancement, radiosensitization, complex damage, Geant4-DNA, Monte Carlo simulation

## Abstract

The use of nanoparticles, in combination with ionizing radiation, is considered a promising method to improve the performance of radiation therapies. In this work, we engineered mono- and bimetallic core-shell gold–platinum nanoparticles (NPs) grafted with poly (ethylene glycol) (PEG). Their radio-enhancing properties were investigated using plasmids as bio-nanomolecular probes and gamma radiation. We found that the presence of bimetallic Au:Pt-PEG NPs increased by 90% the induction of double-strand breaks, the signature of nanosize biodamage, and the most difficult cell lesion to repair. The radio-enhancement of Au:Pt-PEG NPs were found three times higher than that of Au-PEG NPs. This effect was scavenged by 80% in the presence of dimethyl sulfoxide, demonstrating the major role of hydroxyl radicals in the damage induction. Geant4-DNA Monte Carlo simulations were used to elucidate the physical processes involved in the radio-enhancement. We predicted enhancement factors of 40% and 45% for the induction of nanosize damage, respectively, for mono- and bimetallic nanoparticles, which is attributed to secondary electron impact processes. This work contributed to a better understanding of the interplay between energy deposition and the induction of nanosize biomolecular damage, being Monte Carlo simulations a simple method to guide the synthesis of new radio-enhancing agents.

## 1. Introduction

Metal-based nanoparticles have been proposed as highly promising materials to improve medical diagnosis and treatment. The combination of such nanoparticles (NPs) and ionizing radiations has received the great attention of chemists, biologists, and physicists, with the aim to develop new treatment strategies [1]. Smart metal nanoparticles, specifically designed to accumulate in the tumor, actively or passively [2], are able to increase the effect of radiation in a local volume [3,4]. Experimental and theoretical studies [5] demonstrated that the radio-enhancing effect of nanoparticles (commonly referred to as radiosensitization) is initiated by a physical stage, including electron emission and atomic deactivation of the materials.

Gold nanoparticles (Au NPs), in particular, present notable features depending on their size, including specific physical, chemical, and optical properties [6]. Moreover, Au NPs have the ability to increase X-rays image contrast due to their high photoelectric effect cross-section [7]. They are attractive candidates in medical imaging as X-ray contrast agents [7,8] and as adjuvants for radiotherapy [9]. The pioneering work of J.F. Hainfeld and co-workers demonstrated in vivo that Au NPs (1.9 nm—glucose coating) prolonged the life of mice treated with 160 kV X-rays [10,11]. This encouraging result made gold become the most studied agent for a decade.

Beyond Au NPs, other materials, such as platinum, hafnium (NBTXR3) [12], or gadolinium (AGuIX) [13]-based, agents have been extensively studied since they present unique advantages. The two latter materials, developed by the companies Nanobiotix (Paris, France) and NH-TherAguix (Lyon-Grenoble, France), respectively, are currently undergoing clinical trials.

The prediction and modeling of nanoparticles’ effect is an important issue, which aims at developing an adapted treatment planning system (TPS). It has been shown that the effect of nanoparticles cannot be explained in terms of increasing macroscopic dose deposition. Instead, local perturbations must be taken into account to explain the amplification of radiation effects [5,14]. In order to correlate the nanoscale effect and biological damage, Monte Carlo track structure codes have been extensively applied to investigate parameters, such as the beam quality and energy, particle size, concentration and spatial distribution, and secondary electron generation, which are presumed to govern the radiation enhancing properties of Au NPs [5,15,16]. Numerical simulations have been successfully performed with plasmids [17,18] and DNA bases [19], as well as living cells [20,21,22]. Simulation studies performed by S.J. McMahon and co-workers assessed the impact of the nanoparticles’ elemental composition on the radio-enhancement [23]. Their studies showed that gold is not the only heavy atom to consider; in fact, the material has a limited impact on clinical megavoltage (MeV) radiation. They rather found that dose enhancement—due to the presence of metallic nanoparticle—is a complex process at the microscopic level, which requires further investigation. Finding the optimum metal-containing nanoparticle to be efficiently activated by MeV X-rays remains a challenge. The overall radio-enhancement effect cannot be only expected from the material absorption coefficient; it is necessary to better understand the contribution of absorption edges, material density, and Auger electron emission.

This work aimed to evaluate the efficacy of novel ultra-small nanoparticles composed of two metals—gold and platinum—to enhance radiation effects. These bimetallic gold–platinum nanoparticles (Au:Pt-PEG NPs) were chosen because of their advantageous electronic properties, which were used before to enhance catalysis and electrocatalysis [24,25,26]. These properties are related to specific structural and electronic interactions between the two metals. In this work, the nanoparticles were specifically produced for biomedical applications. In this perspective, these nanoparticles were coated with poly (ethylene glycol) (PEG), a ligand widely used in nanomedicine, which is non-toxic and has been approved by the FDA and the EU for internal consumption [27]. It also confers high colloidal stability and biocompatibility, and reduces opsonization (plasma protein adsorption), increasing NPs circulation time and tumor accumulation. The nanoparticles used for this study were synthesized by a unique method, a “green and sterile” technique based on radiolysis. This method, previously developed to produce nano colloids for catalysis [28,29], was adopted by the group from the Institute of Molecular Sciences of Orsay (ISMO) to generate nanoparticles for biomedical applications (French Patent Application: *Synthèse par chimie verte de nanoparticules de platine biocompatibles,* FR1900008). The efficiency of Au:Pt-PEG NPs, to amplify radiation effects, was performed by quantifying the nanosize biodamage induced by γ-rays through the quantification of complex lesions (DSBs, double-strand breaks) induced in plasmid DNA used as nano-bioprobe. The effect of Au:Pt-PEG NPs was compared to the effect of monometallic gold nanoparticles (Au-PEG NPs). In parallel, we simulated the physical processes and compared to the experimental work. 

In order to numerically assess the molecular damage, such as the number of double-strand breaks that can be compared to the experimental data, Monte Carlo simulations were carried out using the Geant4-based architecture for medicine oriented simulation tool (GAMOS) [30]. This was done by following a two-step process. First, secondary electrons generated by the nanoparticles were investigated by using PENELOPE (penetration and energy loss of positrons and electrons) physics, which includes the low energy electromagnetic physics models down to 250 eV. The second step was to superimpose these secondary electrons to the protein of interest and to assess the biomolecular damage induced by the nanoparticles. This last step was made possible with the Geant4-DNA simulation program, an extension of the Geant4 Monte Carlo simulation tool. The advantage of Geant4-DNA is the ability to upload atomic coordinates of any biomolecule from the protein data bank, thanks to the PDB4DNA program developed by E. Delage and co-workers [31]. The protein structure and its function are co-dependent; therefore, a realistic structure is mandatory to simulate the biological environment. Using the PDB4DNA tool means more realistic simulation as the biomolecule structure is based on transmission electron microscopy or X-ray crystallography, which gives an atomistic spatial positioning of the entire protein. The plasmid section available in the protein data bank is the beta-lactamase and was used as the molecular model in our simulation. Direct effects, due to electrons, and indirect effects, due to radicals, have been studied both experimentally and numerically to better understand the origin of nanoparticles radio-enhancement.

We reported here the experimental and theoretical evidence of the radiation enhancing properties of PEGylated bimetallic Au:Pt NPs in comparison with monometallic Au NPs. This work provided new insights into optimal nanoparticle design to achieve maximum efficacy in nanoparticle-aided radiotherapy.

## 2. Results

### 2.1. Characterization of Nanoparticles

**Figure 1:**
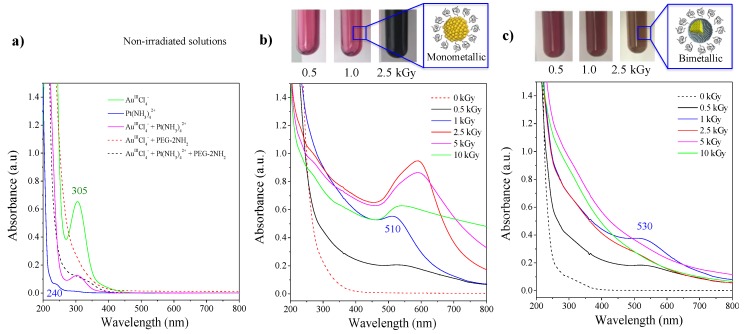
UV-Vis absorption spectra of aqueous solutions containing (**a**) ${\mathrm{Au}}^{\mathrm{III}}{\mathrm{Cl}}_{4}^{-}$ (5 × 10^−4^ mol.L^−1^) and ${\mathrm{Pt}}^{\mathrm{II}}{\left({\mathrm{NH}}_{3}\right)}_{4}^{2+}$ (5 × 10^−4^ mol.L^−1^) separately, mixed together, or mixed with PEG-2NH_2_, (**b**) ${\mathrm{Au}}^{\mathrm{III}}{\mathrm{Cl}}_{4}^{-}$ (10^−3^ mol.L^−1^) mixed with PEG-2NH_2_ before and after irradiation at different doses, and (**c**) ${\mathrm{Au}}^{\mathrm{III}}{\mathrm{Cl}}_{4}^{-}$ (5 × 10^−4^ mol.L^−1^), ${\mathrm{Pt}}^{\mathrm{II}}{\left({\mathrm{NH}}_{3}\right)}_{4}^{2+}$ (5 × 10^−4^ mol.L^−1^) mixed with PEG-2NH_2_, before and after irradiation at different doses. Inserts: images of solutions resulting from 0.5, 1.0, and 2.5 kGy irradiation doses. PEG, polyethylene glycol.

UV-visible absorption spectroscopy revealed ligand-to-metal charge transfer (LMCT) bands of each precursor. In Figure 1a, the peak observed around 305 nm corresponded to the LMCT of ${\mathrm{Au}}^{\mathrm{III}}{\mathrm{Cl}}_{4}^{-}$ ions [32,33], while the low-intensity peak close to 240 nm corresponded to the LMCT of ${\mathrm{Pt}}^{\mathrm{II}}{\left({\mathrm{NH}}_{3}\right)}_{4}^{2+}$ ions [34]. The absorption spectrum of the ${\mathrm{Au}}^{\mathrm{III}}{\mathrm{Cl}}_{4}^{-}$/${\mathrm{Pt}}^{\mathrm{II}}{\left({\mathrm{NH}}_{3}\right)}_{4}^{2+}$ solution displayed the LMCT band of the gold precursor only, while the band of platinum was hidden in the background. The addition of PEG-2NH_2_ to the auric solution (with or without the platinum precursor) induced the disappearance of the 305 nm band, suggesting the formation of an Au^III^-PEG-diamine complex prior to irradiation. There was no evidence of the formation of a transient Pt^II^-PEG-diamine complex.

After exposure of the solution, containing ${\mathrm{Au}}^{\mathrm{III}}{\mathrm{Cl}}_{4}^{-}$ and PEG-diamine (Figure 1b), to 1000 Gy, a band around 510 nm appeared. It corresponded to the surface plasmon resonance (SPR) of small gold nanospheres (<10 nm) [35]. This solution (of red color) was stable for several months when stored at 4 °C. At higher doses of radiation, we observed a redshift in the absorption bands. Along with the new blue reflected color (Figure 1b), it suggested the formation of larger nanoparticles of variable sizes and shapes [36]. This growing effect might indicate that the PEG-2NH_2_ is acting not only as a stabilizer but also as an electron donor, reducing ions adsorbed on the initial nuclei generated by radiolysis and making them grow [28].

By irradiating the bimetallic solution containing ${\mathrm{Au}}^{\mathrm{III}}{\mathrm{Cl}}_{4}^{-}$ (5 × 10^−4^ mol.L^−1^) and ${\mathrm{Pt}}^{\mathrm{II}}{\left({\mathrm{NH}}_{3}\right)}_{4}^{2+}$ (5 × 10^−4^ mol.L^−1^) in the presence of PEG-2NH_2_ (Figure 1c), a rising band at 530 nm was observed, which corresponded to the SPR of gold. This indicated that gold ions were reduced first and aggregated into nanoclusters. At higher doses, the SPR of gold disappeared, and stretched absorption spectra, characteristic of pure colloidal platinum NPs [37], were observed. This spectral behavior was already described [38,39] and corresponded to the formation of bilayered Au_core_/Pt_shell_ NPs with a gold nucleus inside and platinum at the periphery. The complete disappearance of the SPR of gold was observed after exposure to 2500 Gy, suggesting a complete reduction of the auric ions and the formation of a platinum shell. This was confirmed by the color transition from red (gold) to brownish (platinum), as observed in Figure 1c. It is worth mentioning that only bilayered Au_core_/Pt_shell_ particles are formed when Pt^II^ precursor ions are used [38]. The preferential formation of gold clusters is explained by the more noble character and standard REDOX potential of Au (+1.50 eV Au^3+^→Au^0^) compared to Pt (+1.19 eV Pt^2+^→Pt^0^). Indeed, it has been already described that by electron transfer, Pt^II^ slowly reduces Au^III^ into gold clusters and that these clusters then may catalyze the Pt reduction [39]. These core-shell bimetallic gold–platinum nanoparticles (Au:Pt-PEG NPs) diluted in pure water are highly stable when stored at 4 °C. The solution is sterilized in situ by the energetic irradiation.

**Figure 2:**
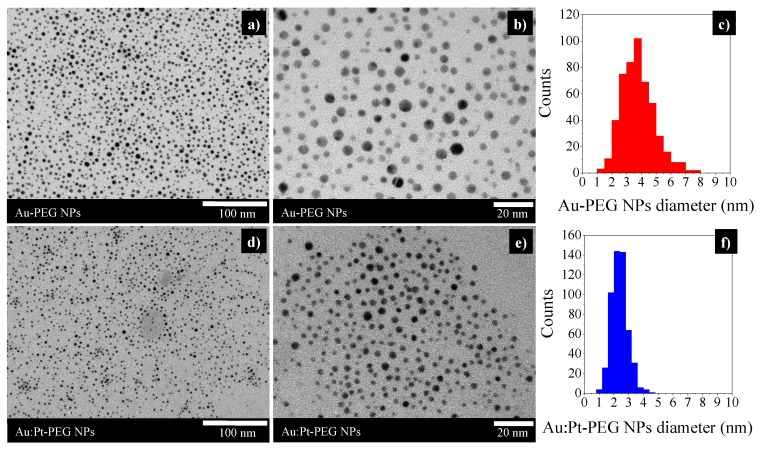
TEM micrographs of Au-PEG NPs (**a**) and Au:Pt-PEG NPs (**d**): scale bar of 1000 nm. Magnified images of Au-PEG NPs (**b**) and Au:Pt-PEG NPs (**e**): scale bar of 20 nm. DLS measurements of Au-PEG NPs (**c**) and Au:Pt-PEG NPs (**f**). NPs, nanoparticles.

TEM micrographs, presented in Figure 2a,b, showed that Au-PEG NPs were homogeneous in size and had a spherical shape with a core diameter of 3.8 ± 1.1 nm (Figure 2c). DLS measurements indicated a hydrodynamic diameter (d_H_) of 10.3 ± 2.7 nm (PdI = 0.290 ± 0.003). The bimetallic Au:Pt-PEG NPs, homogeneous in size and shape, had an average core diameter of 2.4 ± 0.6 nm (Figure 2f) and a d_H_ of 9.3 ± 3.0 nm (PdI= 0.258 ± 0.085).

**Figure 3:**
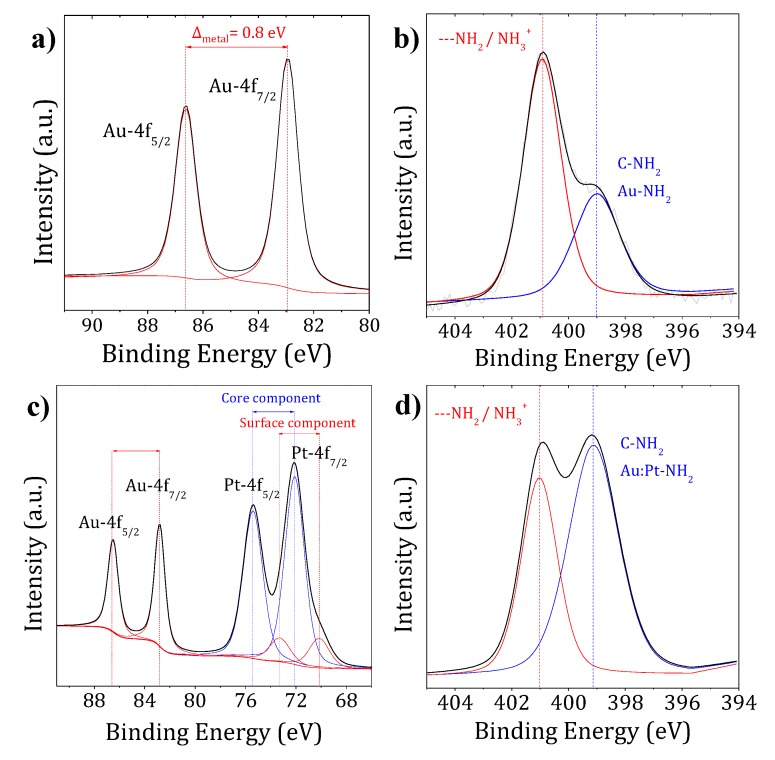
XPS spectra of Au-PEG NPs (**a**,**b**) and Au:Pt-PEG NPs (**c**,**d**) in the regions of Au and Pt-4f binding energies (**a**,**c**) and N-1s binding energies (**b**,**d**).

XPS analysis was used to determine the oxidation state of the metals and the chemical bonding between the ligand and the nanoparticle surface. Au-PEG NPs showed two peaks at 82.9 and 86.6 eV (Figure 3a), which corresponded to the Au-4f_7/2_ and Au-4f_5/2_ spin-orbit components, respectively. The peaks were ~1.2 eV below the binding energy (BE) of the metallic bulk measured as a reference. This result was consistent with reported data of the Au surface state [40,41]. It demonstrated the complete reduction of Au^III^ ions into Au^0^. The C-1s and O-1s peaks observed at 285.9 and 532.2 eV, respectively, corresponded to the PEG-2NH_2_. PEG chains keeping their original chemical structure suggest a monolayer PEG coating configuration [42]. The full width at half maximum (FWHM) values (Table 1) were identical to those of the control (≤0.2 eV), which is an indication of PEG molecules grafted perpendicularly to the nanoparticle surface, as described by other authors [43]. At the N-1s core level, PEG-2NH_2_ had two contributions of around 398.8 and 399.8 eV. These binding energies are characteristic of the primary amine end-group of PEG [40]. For Au-PEG NPs, the N-1s spectrum (Figure 3b) contained two steady peaks at 399.0 and 400.9 eV, which were attributed to non-protonated and protonated amines, respectively. In agreement with other works, the lower binding energy (BE) component was assigned to nitrogen in the pendant amine groups directly bound to gold nanoparticles [44]. The approximate broadening of 36%, from 1.4 to 1.9 eV, confirmed the Au–N binding [43,44]. The component at higher binding energy also suggested the presence of hydrogen-bonded amines (-NH_2_), which might be a consequence of a high graft density [40].

The XPS spectra of Au:Pt-PEG NPs are presented in Figure 3c,d. The observation of four peaks in Figure 3c confirmed the presence of the two metals. A semi-quantitative estimation of the Au:Pt ratio, using the XPS normalized areas, indicated the presence of ~3 times more platinum than gold (22% Au and 78% Pt), in contrast with the synthetic molar ratio used (1:1). However, this XPS ratio was in agreement with a core/shell nanostructure with the aggregation of Pt atoms onto gold seeds. Moreover, the quite high observed Au atomic concentration may be explained as follows: (i) the Pt layer is very thin, so we can assume just a few Pt monolayers as its mean free path is about 1.5 nm, and (ii) it is feasible that some Au remains outside the core-shell structure.

The binding energies at the Au-4f core levels were observed at 82.8 and 86.5 eV, which corresponded to the values for Au^0^ nanoparticles. We observed a higher contribution from the core component of platinum at higher BEs (72.1 and 75.4 eV). This result might suggest not only the presence of Pt^0^ at lower BEs (70.2 and 73.5 eV) but also the presence of Pt–N bonds, which we assumed were the dominant species [45,46,47].

The position of the C-1s and O-1s peaks remained unchanged (Table 1). At the N-1s core level region, bimetallic nanoparticles presented two main contributions (Figure 3d). Similar to Au-PEG NPs, the peak at 399.1 eV corresponded to the direct binding of nanoparticles with the amine end-group of PEG. This was confirmed by the 50% broadening, from 1.4 to 2.1 of the N-1s peak and by the 58% broadening, from 1.2 (Pt^0^ metallic bulk) to 1.9 of the Pt-4f peak. The peak assignments and FWHM for the mono- and bimetallic nanoparticles at the Au-4f, Pt-4f, C-1s, O-1s, and N-1 score levels are summarized in Table 1.

**Figure 4:**
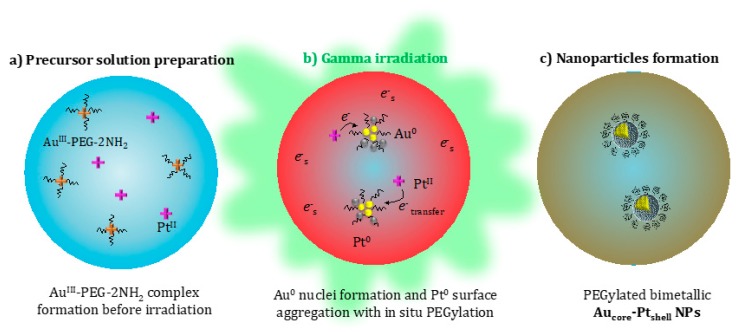
Schematic synthesis of Au:PtPEG-2NH_2_ NPs by the formation of an Au^III^-PEG-2NH_2_ complex (**a**), the radiolytic reduction of metal ions and early electron transfer from Pt to Au (**b**), and formation of Au_core_–Pt_shell_ structured nanoparticles stabilized by PEG-2NH_2_ (**c**).

In summary, Au-PEG NPs consisted of aggregated Au^0^ atoms, while Au:Pt-PEG NPs consisted of Au^0^ atoms aggregated in the core covered by Pt^0^–N species on the shell (Figure 4). Based on the literature, our results suggested that PEG chains were grafted perpendicularly to the NPs’ surface and that amine groups were covalently bonded to nanoparticles.

It is noteworthy to mention the novelty and originality of this one-step “green” and 100% rate synthetic method recently patented (French Patent Application FR1900008) that allows producing biocompatible nano-agents embedded in PEG derivatives widely used in nano-drugs formulations, thanks to their accumulation by enhanced permeability and retention (EPR) effect [48]. Particularly, the size and surface chemistry of nanoparticles determine their accumulation and circulation in the bloodstream [13,49]. Hence, these mono- and bimetallic systems have suitable physicochemical properties (3 nm core size and hydrodynamic diameter of about 10 nm) that allow predicting EPR effect and in vivo tumor penetration in the perspective of cancer treatment through radio-enhancement.

### 2.2. Effect of Nanoparticles on Complex Biodamage Induced by Radiation

**Figure 5:**
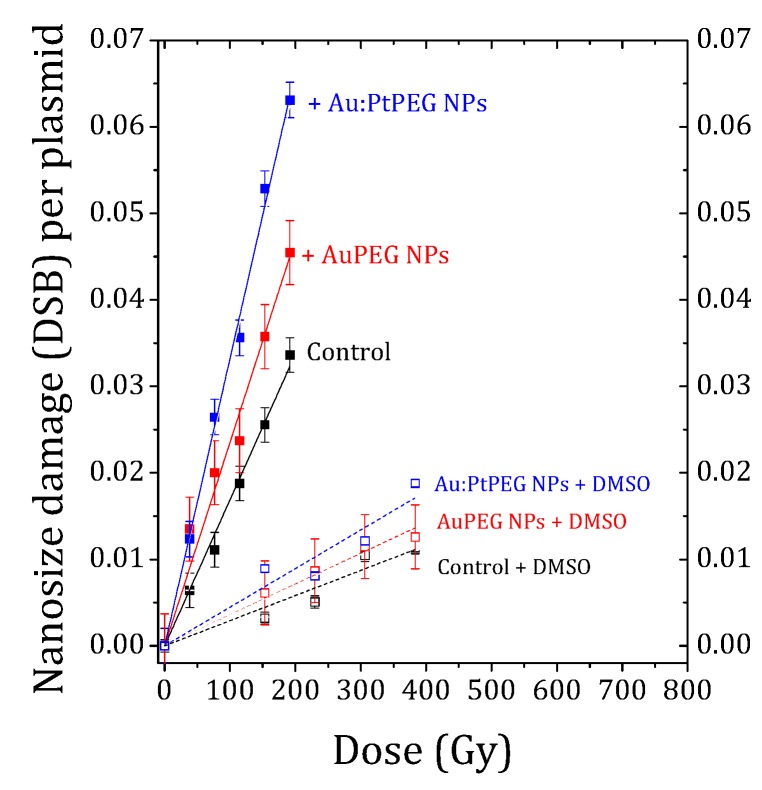
The number of complex damage in the control (■) and samples in the presence of Au-PEGNPs (■) and Au:Pt-PEG NPs (■) as a function of the irradiation dose. The average number of nanosize damage when DMSO is added to the control (□) and samples loaded with Au-PEG NPs (□) and Au:Pt-PEG NPs (□).

The average number of complex biodamage (namely 2 nm DSBs) induced in NPs free samples (control) and in samples which were in the presence of Au-PEG NPs or Au:Pt-PEG NPs were quantified for different radiation doses. The results are reported in Figure 5.

The number of nanosize breaks (DSBs) per plasmid induced in control, and, with NPs, increased linearly with the irradiation dose. The induction of biodamage was strongly amplified in the presence of nanoparticles. More interestingly, the effect was found stronger with bimetallic NPs than with monometallic gold NPs with the same number of metal atoms. 

The efficiency of the mono- and bimetallic nanoparticles to amplify the induction of molecular damage was quantified by using the amplification factor (AF_DSB_), defined as:

(1) $${\mathrm{AF}}_{\mathrm{DSB}}=\frac{Y_{\mathrm{DSB}}\;\mathrm{NPs}-Y_{\mathrm{DSB}}\;\mathrm{Control}}{Y_{\mathrm{DSB}}\;\mathrm{Control}}\times 100$$

The damage yield (Y_DSB_) is defined as the number of DSBs induced per plasmid and per Gy. It corresponds to the slope of the dose-response curves. Table 2 enlists the damage yields and corresponding AFs for NP-free and NP-loaded samples.

The results showed that the yield of nanosize biodamage was strongly enhanced in the presence of PEGylated mono- and bimetallic NPs. The amplification effect of Au-PEG NPs was close to 34%, while the effect of bimetallic nanoparticles was remarkably higher (close to 90%). 

Stated shortly, this experiment showed that Au:Pt-PEG NPs were better radio-enhancers than Au-PEG NPs. We also observed that the induction of nanosize breaks sharply decreased in the presence of DMSO (dotted lines, Figure 5). This confirmed the major role of water radicals in the induction of biodamage, as shown elsewhere [50,51,52]. We evaluated the role of ^•^OH radicals in the induction of nanosize biodamage close to 80%. Stated differently, Au-PEG NPs and Au:Pt-PEG NPs amplified by about 20% the induction of nanosize breaks through other interactions (not solvent-mediated). This effect was in agreement with previous studies performed with platinum and gadolinium-based nanoparticles activated by photons or by ions [50,51,52].

### 2.3. Monte Carlo Simulations

The results obtained using Monte Carlo simulations, to evaluate the contributions of physical processes in the amplification of the radiation-induced nanosize biodamage, trigged by mono- and bimetallic NPs, are presented below.

First of all, we calculated the macroscopic doses deposited by the Cobalt-60 γ-rays source in NPs embedded in a spherical volume of water (water sphere). Table 3 shows the average amount of dose defined as the energy in Joules deposited by unit mass in kilograms (Gy [=] J.Kg^−1^), deposited in the water sphere containing NPs exposed to Cobalt-60 irradiation. For a better understanding of the radiation enhancement, we separated the doses deposited in nanoparticles’ cores and shells. The cores did not change the global metallic composition of both systems. However, the Au shell was used for the monometallic structure and a Pt shell for the bimetallic structure. For both core-shell structures, monometallic Au_core_/Au_shell_ and bimetallic Au_core_/Pt_shell_, the obtained data indicated that the assessment of dose within the shells did not allow a differentiation of the nanostructure effect.

On a second step, we assessed the type of interaction taking place for both metallic nanostructures, ionization by secondary electrons, Compton scattering, photoelectric effect, and Coulombic interaction (also called multi scattering) during secondary electrons scattering. Multiple scattering dealt with the scattering of a charged particle at each simulation step, computed the mean path length correction, and reported the transport of charged particles: electrons and positrons. The model for multi scattering reproduced Coulomb interactions. 

The interaction type for each nanoparticle is reported in Table 4. The total number of physical processes was, on average, 2.74% higher for Au:Pt NPs compared to Au NPs. This was in agreement with the higher radio-enhancement observed experimentally. It was the first indication that the amplification is triggered by primary interactions. The contribution of electronic ionizations, emitting or not secondary particles, was also higher for the bimetallic system. Therefore, the physical mechanism of radio-enhancement due to photon irradiation might be explained by the production of secondary electrons. These results were in agreement with the observations of S.J. McMahon and co-workers [23] who found that electron impact ionization contributes the most to the energy deposition for a 6 MeV photon beam. The production of Compton electrons, Auger electrons, as well as photoelectrons, contributed mostly to the total energy deposited per ionizing event for an atomic number close to Pt (Z = 78) and then decreased as the atomic number increased up to Z = 80.

Multi-scattering processes were found, on average, slightly higher (2.70%) in the water sphere embedding bimetallic nanoparticles. Compton and photoelectric processes were found at low rates. Based on the cross-section measurements and, as expected, Compton scattering was more important than the photoelectric process for a photon beam of 1.25 MeV (median energy of the Cobalt-60 γ-rays). 

**Figure 6:**
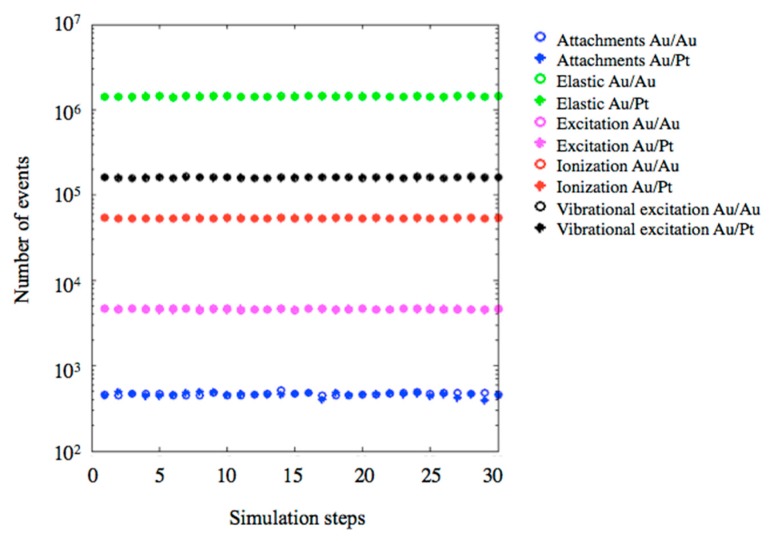
Type and number of scattering events calculated using the Geant4-DNA physical model for the interaction between the cobalt-60 radioactive source (1.25 MeV) and nanoparticles embedded in water. The simulation was performed with 3 × 10^8^ incidents photons divided into 30 steps. The Au_core_/Au_shell_ and Au_core_/Pt_shell_ nanoparticles were simulated separately.

The radiation, induced by bimolecular damage, on the plasmid was evaluated, using Geant4-DNA physics. Figure 6 reports the collisional processes: elastic scattering, electronic excitation, vibrational excitation, ionization, and molecular attachment [53] obtained by Monte Carlo simulation.

The average number of molecular attachments, for the mono- and bimetallic NPs, was found to be 467 and 460, respectively. The average number of ionizations for the Au_core_/Au_shell_ NPs was found to be close to 5.37 × 10^4^, which was slightly higher than the 5.35 × 10^4^ reported for the Au_core_/Pt_shell_ NPs. The average number of vibrational excitations was rather similar for both systems, with 1.61 × 10^5^ and 1.60 × 10^5^ for Au_core_/Au_shell_ and Au_core_/Pt_shell_ NPs, respectively. The same trend was found for other processes: elastic and electronic excitation. The electron direct effect did not indicate any major difference in the radiation damage induced by the two types of nanoparticles. 

DSB was formed by two or more single-strand break (SSB) events on opposite strands. The threshold was set to 10 base pairs. An SSB was counted when an energy deposition greater than 8.22 eV occurred in sugar-phosphate sites (sensitive volumes). This value could be considered as the ionization energy of a DNA molecule. This energy threshold was equivalent to the ionization energy implemented by M.A. Bernal and co-workers [54]. Table 5 shows the results of Geant4-DNA: the energy deposited in the molecule and the number of DSBs or nanosize damage.

The results showed that the number of DSBs caused by Au:Pt NPs and Au NPs, within the uncertainty of the Monte Carlo simulation, were 13.53 and 13.1 breaks per Gy and Gbp. Since the amplification factor depends on the control, we validated the number of DSBs simulated in the absence of nanoparticles by comparing them with available data. As indicated in Table 5, DSBs for the control sample was about nine. This result was consistent with the experimental data reported by U. Klimczak and co-workers [55]. To compare the numerical results with the experimental data, we calculated the AFs of the nanosize biodamage (see Equation (1)). The results reported in the last column of Table 5 show that Au:Pt NPs amplified by 45% the direct induction of nanosize damage. These results were in good agreement with the experimental determination, which was found when suppressing the indirect effects using a radical scavenger, dealing only with secondary electrons from Cobalt-60 γ-rays (Table 2). However, when considering both the direct and indirect secondary electrons, Au:Pt NPs showed a radiation enhancement factor much larger than Au NPs. This indicated that the bimetallic system caused more DSBs, especially at the chemical stage. Another feature that might cause the observed differences but that has not been characterized in the current numerical study is the spatial distribution of energy deposits. 

## 3. Discussion

In this study, we analyzed the ability of Au and Au:Pt nanoparticles to increase the energy deposited by radiation in therapy applications. In light of ongoing research, it was concluded that monometallic nanoparticles composed of a single element were certainly not the most efficient for application in radiotherapy. We developed new nanoparticles composed of a gold core and a platinum shell. These stable sub-5 nm spherical metallic nanoparticles were incubated using a molecular probe. We reported the first experimental evidence that bimetallic nanoparticles, which combine the advantages of its physicochemical and biological properties, were greater radio-enhancers than monometallic nanoparticles. Considering the direct effects due to primary radiation and secondary electrons, and indirect effects due to hydroxyl radicals, we observed an increase of 90% for bimetallic versus 34% for monometallic in the induction of nanosize biodamage, suggesting that bimetallic engineered nanoparticles are more efficient. In order to validate our experimental results, we carried out a numerical simulation using the Geant4-DNA Monte Carlo nanodosimetry code. We evaluated the radio-induced biomolecular damage in a section of the plasmid. Considering exclusively the direct effects caused by primary photons and secondary electrons, we found a radiation enhancement of 45% for bimetallic nanoparticles in very good agreement with the 42% evaluated experimentally for the Au:Pt-PEG NPs. Indeed, Monte Carlo simulations confirmed that the bimetallic NPs had greater radio-enhancing properties than the monometallic NPs. The agreement between our numerically-predicted radiation nanosize damage and the experimental one suggested that the increased molecular damage from Au:Pt NPs did not come from electron direct interactions. This has been also demonstrated in previous experimental studies [17,55]. According to the experimental results, the yield of nanosize damage depends mainly on the chemical stage that takes place after the physical interaction. In the future, it is planned to simulate the chemical phase to analyze the radical effects for a better understanding of the mechanisms, underpinning radiation damage in the presence of nanoparticles. Finally, we demonstrated, based on experiments corroborated by numerical simulations, that bimetallic nanoparticles with a gold core and platinum shell possessed higher radio-enhancing properties than gold monometallic nanoparticles. Our results opened new avenues for optimal material combination to design more efficient nano-agents.

## 4. Materials and Methods 

### 4.1. Experimental Section

#### 4.1.1. Materials

Tetraamineplatinum (II) chloride (Pt(NH_3_)_4_Cl_2_, 99%) and tetrachloroauric (III) acid (HAuCl_4_, 99.99%) from Sigma–Aldrich, (St. Quentin Fallavier Cedex, France) were used as metallic precursor salts. Homobifunctional poly (ethylene glycol) diamine (PEG-2NH_2_, Mw= 2000 g.mol^−1^) from Sigma–Aldrich (St. Quentin Fallavier Cedex, France), was used as a stabilizer and biocompatible coating agent. High purity nitrogen (N_2_) was purchased from Air Liquid, (Antony, France). The pBR322 plasmid DNA (4361 base pairs, 2.83 × 10^6^ Da) from Life Technologies SAS (Saint Aubin, France) was used as the nano-biomolecular probe. All the chemicals were used as received without further purification. The named ultrapure water is Milli-Q Millipore (MQ: 18.2 MΩ cm at 20 °C).

#### 4.1.2. Radiolytic Synthesis

The mono- and bimetallic gold–platinum nanoparticles were obtained by the gamma irradiation-assisted method described in the French Patent Application FR1900008. In summary, nanoparticles were synthesized in water without the addition of reducing agents at M_T_ = 10^−3^ mol.L^−1^ (M_T_, total metal concentration) and the optimal PEG-2NH_2_:M_T_ molar ratio of 50. During radiolysis, the solvated electrons and H^•^ radicals reduce the metal precursors homogeneously, leading to stable metal NPs that are highly homogeneous in size and shape [56]. The bimetallic nanoparticles were prepared with a gold:platinum molar ratio of 1. Various aqueous solutions containing the metal ions and the PEG-2NH_2_ were deaerated by nitrogen bubbling and irradiated with a panoramic ^60^Co gamma source (γ-rays of 1.17 and 1.33MeV, LET = 0.2 keV.µm^−1^) at increasing doses, from 0 kGy up to 10 kGy, with a dose rate of ca. 95.5 Gy.min^−1^.

#### 4.1.3. Physico-Chemical Characterization

Ultraviolet-visible spectroscopic measurements were performed to follow the reduction of the metal ions (Au^III^ and Pt^II^) and nanoparticles formation in a Agilent 8453 UV-visible spectroscopy system, equipped with a deuterium-discharge lamp for the UV region (190–370 nm) and a tungsten lamp for the visible and short wave near-infrared (370–1100 nm). Observed peaks at around 370 nm are small artifacts commonly produced when the electromechanically actuated shutter allows light to pass through the sample by changing the emission lamp. The hydrodynamic diameter (d_H_) of nanoparticles was determined by dynamic light scattering (DLS) at room temperature using a Zetasizer Nano-ZS (laser He–Ne, 633 nm) from Marven Instrument Ltd. (Orsay Cedex, France). The d_H_ represents the mean of three independent NPs batches. The metal core size and shape were determined by TEM with a JEOL 100CXII TEM at an accelerating voltage of 100 kV. Drops of fresh PEGylated nanoparticles were deposited on carbon-coated copper grids and observed after natural drying. The nanoparticles size distribution histograms were built by analyzing close to 20-recorded images and 2000 objects with the ImageJ 1.46r software. XPS measurements were performed in a K Alpha XPS spectrometer equipped with a monochromatic aluminum source (Al K_α_, 1486.7 eV) under ultrahigh vacuum (10^−9^ mbar). The XPS analyses were performed by fixing a spot size of 400 µm, which corresponds to an irradiated zone of approximately 1 mm^2^. The hemispherical analyzer was operated at a 0° take-off angle using the Constant Analyzer Energy mode. The survey scans were acquired with a pass energy of 200 eV. The narrow windows were obtained by using a pass energy of 50 eV with a 0.1 eV step. Charge compensation was done by means of a “dual beam” flood gun. The recorded spectra were processed using a peak-fitting routine with Shirley background, symmetrical 70–30% mixed Gaussian–Lorentzian peak shapes to fit C-1s, O-1s, and N-1s, and a Doniach–Sunjic type function to fit the metallic asymmetric peaks. The native PEG-2NH_2_ was measured as a powder (directly from the supplier) and nanoparticles as colloids after drop-casting and natural drying. The results were reproductively obtained over at least three independent NPs synthesis.

#### 4.1.4. Preparation and Analysis of the Biomolecular Samples

The pBR322 plasmid was used as a nano-biomolecular probe. It consists of a circular double-stranded DNA of 4361 base pairs (2.83 × 10^6^ Da). It is supplied in solution at 0.5 μg.μL^−1^ in a TE buffer consisting of 10 mmol.L^−1^ Tris-HCl (pH = 7.6) and 1 mmol.L^−1^ ethylenediaminetetraacetic acid (EDTA). Its native morphology consists of the supercoiled conformation by more than 95% and ca. 5% of the relaxed conformation that corresponds to the plasmid with single-strand breaks (SSB). The linear conformation that corresponds to the plasmid with two breaks in two strands separated by less than ten base pairs (DSB) is not present in the native product.

The samples were prepared, as described elsewhere [50,52]. In brief, each sample contained 500 ng of DNA (1 µL) in 12.3 µL of TE buffer. The NPs were diluted to reach a final metal concentration of 4.23 × 10^−5^ mol.L^−1^ and added 1 h prior to irradiation (2.4 µL). The final sample volume was adjusted to 18 µL with ultrapure water. The molar ratio used corresponded to ca. the addition of one NP per 2 plasmids (considering a 3 nm NP with about 1000 metal atoms). Control samples were prepared by adding 4.7 μL of ultrapure water to the plasmid instead of nanoparticles. Dimethylsulfoxide (DMSO) was added to some samples at a final concentration of 1.0 mol.L^−1^. The samples were irradiated at doses ranging from 0 up to 500 Gy, with a dose rate of ca. 8.4 Gy/min. The dose rate of the panoramic ^60^Co gamma source at the experimental conditions was determined by Fricke dosimetry. After irradiation, the radiation-induced damage was analyzed by agarose gel electrophoresis. The migration was performed at 80 V and 4 °C. DNA was stained after migration in a 0.02% ethidium bromide aqueous bath and rinsed with water prior to imaging. After staining, the lines corresponding to plasmids with different conformations were revealed under UV light at 302 nm and recorded with a CCD camera. The densitometry was performed by using the Image Quant 5.0 software. The yields of double-strand breaks (mDSB, nanosize damage) were calculated, as described elsewhere [50,52]. Briefly, the densitometry was normalized for DSB (nanosize damage) as follows:

(2) Total=1.47×S+R+L

(3) $$L^{\prime }=\frac{L}{\mathrm{Total}}$$

The radio-induction yields of DSB were calculated according to a Poisson law that statistically fits low probabilities [57] by:

(4) $$\mathrm{DSB}\;\mathrm{yield}\;\left(\text{breaks per plasmid}\right)=\frac{L^{\prime }}{1-L^{\prime }}$$

The dose-response curves were thus obtained by plotting the average DSB yields as a function of the irradiation doses. The error bars represented the standard derivation of the independent triplicates. The global damage yield (Y_DSB_), defined as the number of DSBs induced per plasmid and per Gy, corresponded to the slope of the dose-response curves. Hence, the fitted slope was used to analyze the efficiency of nanoparticles to amplify radiation molecular damage. 

### 4.2. Theoretical Section

#### 4.2.1. Calculation of the Platinum Shell Thickness

The platinum shell thickness *T* was derived from the XPS measurements using an analytical method [58,59]. This method is rather simple and depends only on two parameters: (i) the average electron attenuation length *L_film_* of the photoelectrons, and (ii) the overlayer *R*. Thus, *T* is given by:

(5) $$T=L_{film}\cos\left(\theta \right)\ln\left(1+R\right)$$

where *R* = 3 nm is the mean core diameter, and *θ* = 0° is the scattering angle under experimental conditions. The *L_film_* values are 1.02 nm and 1.05 nm, respectively, for gold and platinum, which are based on the NIST database [59]. Therefore, for the bimetallic system, the platinum shell thickness was found to be 1.4 nm, with an uncertainty of 4% [60]. In order to set up the geometry in the Monte Carlo program, both types of nanoparticles were made of 2 nm shells thickness and 3 nm cores diameter. This approximation ensured to account the uncertainties of the analytical determination.

#### 4.2.2. Monte Carlo Simulations

Simulations were carried out using the Geant4-based architecture for medicine oriented simulations tool (GAMOS) [30] 5.1.0 version over the Geant4 10.03 version. Data analysis was performed with the ROOT software developed by the Conseil Europeen pour la Recherche Nucleaire (CERN). The visualization of the geometry was performed using the view3dscene (https://castle-engine.io/view3dscene.php) open-source software under the GNU free documentation license that also served as a verification tool of the simulation program. The visualization of the physical processes was carried out using a homemade application, which was originally designed for the low energy particle track structure code (LEPTS) nanodosimetry software [61]. Simulations were carried out at the *Centro de Investigaciones Energéticas, Medioambientales y Tecnológicas* (CIEMAT) computer cluster. The simulations were performed following the method described by Y. Lin and co-workers [62], which divides simulation of nanoparticle under irradiation into two parts: (i) Geant4-Penelope to assess the radiation-nanoparticle interactions, and (ii) Geant4-DNA to evaluate the molecular damage induced by radiation using the phase space of the previous step. The materials properties (isotope, density, pressure, and temperature) were based on the NIST default reference values. To elucidate the role of platinum in enhancing the effects of radiation, we reproduced the NPs simulation geometry, but now by changing the material of the shell. An approximation of 191 nanoparticles, which resemble the experimental metal concentration of 4.23 × 10^−5^ mol.L^−1^, was randomly distributed inside a microscopic water sphere of 50 µm^3^ following a homogenous distribution. The water phantom was set in the center of a 5 × 5 × 5 µm^3^ cubic volume filled with air that mimicked the source location. The γ-rays were simulated as two mono-energetic peaks of 1.33 and 1.17 MeV having a conical shape and an angular width of 1.14° each.

#### 4.2.3. Geant4-Penelope

To track the secondary electrons, we applied the Geant4-Penelope physics, which includes the low energy electromagnetic physics models based on PENELOPE (penetration and energy loss of positrons and electrons). This model includes secondary electrons, which not only carry out most of the energy of the primary beam but also play an important role in inducing radiation damage [63,64]. In our simulation, the energy threshold at which electrons were tracked using Penelope was limited to 250 eV [65,66]. GAMOS scoring functions were used to assess the macroscopic dose in the water sphere, the shells, and the cores of nanoparticles. PENELOPE served to model the interaction with the water without the plasmid. The next step was to model the interaction of the radiation with the plasmid. This was made with Geant4-DNA.

#### 4.2.4. Geant4-*DNA*

**Figure 7:**
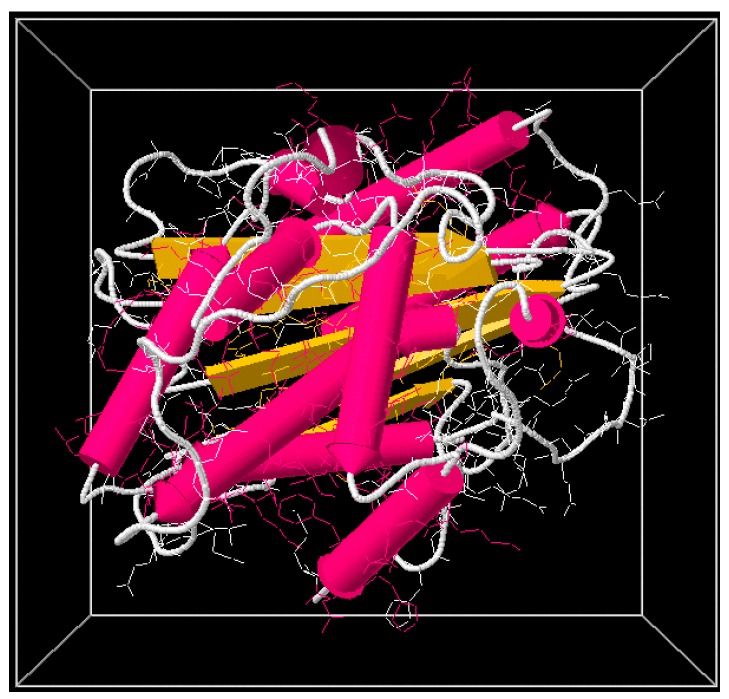
The illustration of the beta-lactamase section of the pBR322plasmid DNA.

The second step of the simulation was assumed as a primary source, previously recorded phase space (energy and momentum distribution of secondary electrons) at the center of the water phantom, in order to simulate the damage induced to the biomolecule. The simulated biomolecule was the beta-lactamase section of the pBR322 plasmid. Coordinates of the biomolecule were extracted from the 1btl.pdb file [67] of the Protein Data Bank (http://www.rcsb.org/pdb/). A representation of the backbone structure of the beta-lactamase is shown in Figure 7 in strand mode using the Matlab version 7.10.0 Molecule Viewer application, molviewer from MathWorks [68].

Geant4-DNA has been developed for the study of radiosensitization in radiotherapy using NPs but also for investigating radiation effects from various radiation qualities, such as hadron therapy and photon therapy. This extension of Geant4 provides physical processes and models able to describe particle interactions in liquid water at the nanometer scale. Indeed, it was suitable for our study, assuming that the molecule has similar cross-section as water [69]. The electron processes within Geant4-DNA include ionization, electronic excitation, elastic scattering, and two sub-excitation processes: vibrational excitation and molecular attachment [69]. The molecular attachment cross-section is based on experiments performed by C.E. Melton [70], while the cross-section for vibrational excitation is derived from M. Michaud and co-workers measurements, with a correction taken into account for the liquid phase [20,71]. Other inelastic interactions, comprising both ionization and electronic excitation, are derived from the model of the dielectric response function of liquid water [72,73].

## 5. Patents

French Patent Application FR1900008 “Synthèse par chimie verte de nanoparticules de platine biocompatibles”.

## Figures and Tables

**Table 1:** Summary of the obtained XPS data BE (binding energy), FWHM (full width at half maximum), and the assignment from the enlisted references of ${\mathrm{Au}}^{\mathrm{III}}{\mathrm{Cl}}_{4}^{-}$, ${\mathrm{Pt}}^{\mathrm{II}}{\left({\mathrm{NH}}_{3}\right)}_{4}^{2+}$, Au^0^ solid, Pt^0^ solid, polyethylene glycol (PEG)-2NH_2_, Au-PEG NPs (nanoparticles), and Au:Pt-PEG NPs.

Compound	Atomic Core Level	BE (eV)	FWHM (eV)	Assignment
${\mathrm{Au}}^{\mathrm{III}}{\mathrm{Cl}}_{4}^{-}$ *	Au-4f_7/2_	87.8	1.3	Au^III^
	Au-4f_5/2_	91.5	1.3	Au^III^
${\mathrm{Pt}}^{\mathrm{II}}{\left({\mathrm{NH}}_{3}\right)}_{4}^{2+}$	Pt-4f_7/2_	73.0	1.7	Pt^II^
	Pt-4f_5/2_	76.3	1.7	Pt^II^
	N-1s	400.2	1.6	Pt-NH_3_^+^
Au^0^ solid	Au-4f_7/2_	84.1	0.9	Au^0^
	Au-4f_5/2_	87.8	0.9	Au^0^
Pt^0^ solid	Pt-4f_7/2_	71.1	1.2	Pt^0^
	Pt-4f_5/2_	74.5	1.2	Pt^0^
PEG-2NH_2_	C-1s	285.9	1.3	C–O and C–N
	O-1s	532.4	1.4	C–O–H
				C–O–C
	N-1s	398.8	1.4	C–N
	N-1s	399.8	1.4	C-NH_2_
Au-PEG NPs	Au-4f_7/2_	82.9	0.8	Au^0^
	Au-4f_5/2_	86.6	0.8	Au^0^
	C-1s	285.9	1.2	C–O and C–N
	C-1s	284.4	1.0	C–C
	O-1s	532.2	1.3	C–O–H
				C–O–C
	N-1s	399.0	1.9	C-NH_2_
				Au-NH_2_
	N-1s	400.9	1.6	---NH_2_
Au:Pt-PEG NPs	Au-4f_7/2_	82.8	1.0	Au^0^
	Au-4f_5/2_	86.5	1.0	Au^0^
	Pt-4f_7/2_ surf.	70.2	1.9	Pt^0^
	Pt-4f_5/2_ surf.	73.5	1.9	Pt^0^
	Pt-4f_7/2_ core	72.1	1.8	Pt–N
	Pt-4f_5/2_ core	75.4	1.8	Pt–N
	C-1s	285.9	1.2	C–H and C–N
	C-1s	284.3	0.9	C–C
	O-1s	532.2	1.3	C–O–C
	N-1s	399.1	2.1	C-NH_2_
				Au:Pt-NH_2_
	N-1s	401.0	1.5	---NH_2_/NH_3_^+^

* the HAuCl_4_ compound was reduced under XPS analysis (Au4f^7/2^ at 85.3 eV assigned to Au^I^).

**Table 2:** Yields of nanosize biodamage (m_DSB_) induced by radiation in the control and NP-loaded samples. Amplification factors (AFs) of mono- and bimetallic NPs and the contribution of water-mediated effect (OH effect).

Sample	m_DSB_ (×10^−5^) (Breaks Per Plasmid Per Gy)	AF_DSB_ (%)	OH Effect (%)
Control	17.47 ± 0.51	-	-
Au-PEG NPs	23.49±0.94	34 ± 2	-
Au:Pt-PEG NPs	33.18 ± 0.54	90 ± 2	-
Control + DMSO	2.91 ±0.22	-	83 ± 1
Au-PEG NPs + DMSO	3.56 ± 0.13	22 ± 4	85 ± 1
Au:Pt-PEGNPs + DMSO	4.14 ± 0.36	42 ± 2	86 ± 1

**Table 3:** Dose deposition to sensitive targets: water sphere embedding nanoparticles, shell, and core of mono- and bimetallic nanoparticles.

Section	Dose (Gy)	
	Monometallic Au_core_/Au_shell_	Bimetallic Au_core_/Pt_shell_
Water sphere	47.8	47.9
Shell	143.2	143.9
Core	375.8	383.4

**Table 4:** Physical processes from the Geant4-Penelope model induced in mono- and bimetallic NPs activated by Cobalt-60 γ-rays and the corresponding electron secondary spectrum. The simulation steps of 3 × 10^8^ were used to produce the output results.

Physical Process	Monometallic Au_core_/Au_shell_	Bimetallic Au_core_/Pt_shell_
Electronic ionization from electrons	191	204
Direct electron impact ionization from electrons	50	59
Multi Coulomb scattering from electrons	4,874,768	5,008,513
Compton scattering from photons	35	36
Photoelectric effect from photons	9	7
Total	4,875,053	5,008,819

**Table 5:** Nanosize damage (DSBs) from Geant4-DNA simulation based on the energy deposited (MeV) in the beta-lactamase section of the pBR322 plasmid.

System	Energy Deposition (MeV)	DSBs (Gy^−1.^Gbp^−1^)	AF (%)
Beta lactamase	1.075	9.32 ± 0.05	-
+Au NPs	0.913	13.10 ± 0.03	40 ± 1
+Au:Pt NPs	0.916	13.53 ± 0.02	45 ± 1

## Abbreviations

AF_DSB_	Amplification factor of radio-induced double-strand breaks (nanosize biodamage)
Au NPs	Gold nanoparticles
Au-PEG NPs	PEGylated gold nanoparticles
Au:Pt-PEG NPs	PEGylated bimetallic Au_core_/Pt_shell_ nanoparticles
BE	Binding energy
d_H_	Hydrodynamic diameter
DMSO	Dimethylsulfoxide
DSBs	Double-strand breaks
EDTA	Ethylenediaminetetraacetic acid
EPR	Enhanced permeability and retention effect
FWHM	Full width at half maximum
GAMOS	Geant4-based architecture for medicine oriented simulations tool
LEPTS	Low energy particle track structure code
LMCT	Ligand-to-metal charge transfer
mDSB	yields of double-strand breaks (nanosize biodamage)
PdI	Polidispersity index
PEG	Poly (ethylene glycol)
PEG-2NH_2_	Homobifunctional poly (ethylene glycol) diamine
PENELOPE	Penetration and energy loss of positrons and electrons
SPR	Surface plasmon resonance
SSB	Single-strand breaks
TPS	Treatment planning system
XPS	X-ray photoelectron spectroscopy
